# Former Foodstuff-Based Diets on the Quality and Sensory Properties of Eggs from Laying Hens

**DOI:** 10.3390/ani16203220

**Published:** 2026-10-13

**Authors:** Luisa Zaniboni, Niko Gioacchino Zeni, Cristina Tognoli, Sabrina Ratti, Edda Mainardi, Raffaella Rossi

**Affiliations:** 1 Department of Veterinary Medicine and Animal Science, Università Degli Studi di Milano, Via Dell’Università 6, 26900 Lodi, Italy; luisa.zaniboni@unimi.it (L.Z.); cristina.tognoli8@gmail.com (C.T.); edda.mainardi@unimi.it (E.M.); raffaella.rossi@unimi.it (R.R.); 2 Independent Researcher, 20100 Milano, Italy; sabrinaratti@hotmail.com

**Keywords:** egg quality, laying hens, former foodstuff products, sensory analysis, sustainability

## Abstract

In recent years, former food products (FFPs) have been gaining attention as alternative feed ingredients for more sustainable production systems. This study evaluated the impact of including FFPs on the quality of eggs produced by laying hens. A total of 180 Lohmann Brown^®^ hens were divided into three groups and fed a control diet (CON), a diet containing 20% FFPs (T20), and a diet containing 40% FFPs (T40). Eggs, collected between 31 and 33 weeks of age, were evaluated for physical, chemical, and sensory characteristics. Egg weight, shape index, yolk and albumen weight, and edible portion were comparable (*p* > 0.05) among the groups. Moreover, shell thickness, shell weight, and shell proportion were unaffected (*p* > 0.05) by dietary treatments. Yolk color was affected by FFP inclusion, with reduced (*p* < 0.05) yellowness in the supplemented groups, likely due to the lower dietary xanthophyll content resulting from the partial replacement of corn. The inclusion of FFPs had limited effects on the sensory quality of the eggs, with differences (*p* < 0.05) in yolk color and albumen texture. These findings highlight the potential of FFPs as a viable feed ingredient in laying hen diets. Further research is needed to confirm their applicability in different diets and farming conditions.

## Simple Summary

The use of former foodstuff products (FFPs) is key to improving the sustainability of livestock production. Consisting of food that is no longer intended for human consumption for commercial or logistical reasons, FFPs can be recycled as feed ingredients and contribute to circular economy strategies. FFPs are characterized by high concentrations of digestible carbohydrates and energy, making them valuable ingredients in poultry feed. In the poultry sector, however, information is limited and sometimes conflicting, particularly with regard to egg quality and sensory characteristics. This study aimed to evaluate the effects of different dietary levels of FFPs on egg quality in laying hens. The physical, chemical, and sensory characteristics of the eggs were assessed. Slight differences in yolk color and sensory attributes were observed among dietary treatments. These results suggest that FFPs can be included in laying hen diets with limited effects on egg quality parameters, supporting their potential use as a sustainable feed ingredient.

## 1. Introduction

Global demand for sustainable animal production systems is increasing due to concerns over climate change and resource availability. In the poultry sector, in particular, the use of conventional feed ingredients, such as corn and soy, exert significant pressure on land, water, and energy resources, increasing interest in alternative feed sources aligned with the principles of the circular economy [1].

In this context, the use of agro-industrial by-products and food waste offers a promising approach to improve the sustainability of livestock production while reducing the environmental impact of producing feed and treating food waste [2]. However, the availability, classification, and regulatory framework relating to the use of these materials as feed ingredients differ significantly among countries. In the United States, for example, food waste materials such as bakery meal are commonly used in animal feeding and are subject to specific federal provisions about their use. In China, by contrast, information on the regulatory framework for the use of food waste as animal feed is more limited [3,4]. These differences highlight that the availability and practical use of former foodstuff products (FFPs) as feed ingredients are strongly influenced not only by their nutritional characteristics but also by national legislation.

From a European perspective, FFPs are food products that have been manufactured in full compliance with EU food legislation for human consumption. However, they are no longer intended for this purpose due to practical or logistical reasons. FFPs mainly comprise by-products from the bakery and confectionery sectors, such as bread, pasta, biscuits, breakfast cereals, cakes, and snacks that maintain high nutritional and safety standards. In detail, according to the definition in the European Union Catalogue of Feed Materials, these are ‘food products that were originally manufactured for human consumption, but which are no longer suitable for the food market due to production errors, production surpluses, or logistical difficulties’. This includes products that do not meet food standards due to incorrect shape, color, taste, labelling or packaging, as well as products past their use-by date, surpluses resulting from discontinued product lines or logistical issues, and unsold products after events [5]. Using these materials in animal nutrition is considered a practical application of the principles of the circular economy, as it enables nutrients and energy that would otherwise be discarded to be reintegrated into the food production chain. This supports the transition towards more sustainable and resource-efficient livestock production systems [2].

The FFPs are characterized by high concentrations of digestible carbohydrates and energy, making them a valuable ingredient in poultry feed, according to the European Feed Manufacturers’ Federation (FEFAC) [6]. Research on the use of FFPs in livestock diets indicates their potential as a sustainable feeding strategy [7]. A recent review reported that FFPs can partially replace cereal grains in the diets of several animal species. Research has focused predominantly on monogastric animals rather than ruminants. In pigs, the inclusion of FFPs in diets has been shown to improve nutrient digestibility and energy supply while maintaining productive performance, highlighting their potential as sustainable alternatives to conventional feed ingredients [8,9,10]. Ruminants have attracted less interest than monogastric animals because the high levels of simple sugars present in FFPs may increase the risk of subacute ruminal acidosis due to rapid fermentation in the rumen, caused mainly by saccharolytic bacteria [11,12].

In the poultry sector, several studies have investigated productive performance and meat quality parameters [13,14,15]. In contrast, information about the use of FFPs and their effects in laying hens is limited and sometimes conflicting, particularly regarding egg quality and sensory characteristics. Moreover, the high variability in FFP composition, related to the included food products, requires further investigation to optimize their use in laying hen diets [9]. However, FFPs are particularly interesting due to their high energy value and digestibility, which may allow the partial replacement of conventional cereal-based ingredients in feed formulations.

Despite the well-documented sustainability benefits of circular feed strategies involving FFPs, consumer attitudes towards environmental issues significantly impact food choices [16]. Previous research suggests that greater environmental awareness may increase consumers’ acceptance of poultry products obtained through circular practices, as reported in studies conducted in European countries [17].

Within this framework, the present study aimed to provide a physical, chemical, and sensory characterization of eggs obtained from laying hen fed diets containing increasing levels of FFPs. This approach was adopted to assess the impact of dietary inclusion of FFPs on the quality attributes of the final product.

## 2. Materials and Methods

### 2.1. Animal Management

A total of 180 Lohmann Brown^®^ laying hens were obtained from Alberici Farm (Castelletto Sopra Ticino, Novara, Italy). The birds were housed in furnished cages (Big Dutchman International GmbH, Vechta, Germany) and managed in accordance with the requirements of Council Directive 1999/74/EC of 19 July 1999 [18], which establishes minimum standards for the protection of laying hens. The trial was conducted at the poultry facility of the Animal Production Centre of the University of Milan in Lodi, Italy. At 19 weeks of age, hens were individually identified using removable leg rings with sequential numbering and randomly distributed among three experimental groups (60 birds per group). Each group was allocated to five cages containing 12 hens each, providing a stocking density of 750 cm^2^ per hen. The cages measured 150 cm in width, 60 cm in depth, and 52 cm in height and were equipped with four automatic nipple drinkers, a linear feeder allowing 12 cm per hen, plastic perches providing 15 cm per hen, and a nest box. Manure was removed through an automatic belt system located beneath the cage floor, while eggs were manually collected from an egg belt positioned under the feeder line outside the cage. After arrival at the facility, hens were allowed 14 days of adaptation period to the new housing conditions. During the trial, birds were kept in a controlled environment with the temperature maintained between 20 and 24 °C and the relative humidity between 50 and 60%. Air exchange was ensured by a positive-pressure forced ventilation system. The photoperiod initially consisted of 10 h of light and gradually increased by 1 h per week until reaching 14 h of light at 23 weeks of age. The entire experimental period lasted 14 weeks. The health status of the hens was checked and recorded twice daily.

### 2.2. Experimental Design

From 21 to 35 weeks of age, hens were assigned to three dietary treatments: a control diet for laying hens in oviposition (CON), the control diet including 20% FFPs (T20), and the control diet including 40% FFPs (T40). The control and experimental diets were prepared by a feed factory (PBA Mangimi s.r.l, Fombio, Lodi, Italy) according to our specifications. The calculated xanthophyll concentrations of the experimental diets were 16.78, 11.38, and 7.68 mg/kg for CON, T20, and T40, respectively, as provided by the feed manufacturer. These values were not analytically determined in the present study. The FFP used in the study was a commercially available product (PRIMO^®^, Regardia s.r.l., Marene, Cuneo, Italy) consisting of bakery by-products, dry pasta, chocolate, and puffed cereals (analyses provided by the producer: Dry matter 88%, crude protein 10.5%; ether extract 10.5%; crude fiber 2.2%; ash 2.2%; starch 40%; potassium 0.39%; sodium 0.3%; chloride 0.29%; phosphorus 0.18%; calcium 0.10%). All experimental diets were formulated to be isoenergetic (CON: 2980 kcal ME/kg; T20: 2979 kcal ME/kg; T40: 2980 kcal ME/kg), isonitrogenous, and to contain the same level of linoleic acid. The ingredients and chemical composition of the experimental diets are presented in Table 1. All diets were provided in crumble form and offered ad libitum once daily, while fresh drinking water was always available.

### 2.3. Sample Collection and Testing

A total of 30 eggs per treatment (6 eggs per cage) were collected weekly from each experimental group for three consecutive weeks during the experimental period, when hens were 31–33 weeks of age. After collecting, the eggs were stored at 4 °C for three days and then allowed to kept for at 4 h ambient temperature before physical analyses (4 eggs per cage). Then, the edible components (yolk + albumen) of the eggs were pooled and homogenized for chemical analyses. Five pools (1 pool/cage/weekly) were stored at −20 °C until the determination of chemical parameters. For sensory evaluation, the eggs (2 eggs per cage) were stored at +4 °C for 6 days (fresh egg category). The sensory evaluation of the eggs was carried out over the three-week period.

### 2.4. Physical Analyses

On samples, the following parameters were determined. Egg weight (EW, g) was determined using a digital precision scale (GP3202, Sartorius Srl, Varedo, Italy). Egg diameter (D, equatorial axis) and length (L) were measured with a micrometer (Digital Stainless Hardened, Sordalab, Étampes, France), and the shape index was calculated as D/Lx00 [19]. Based on egg dimensions, egg volume (V = 0.525 × L × D^2^; cm^3^), surface area (SA = [0.9658 × D/L + 2.1378] × L × D; cm^2^), and the area/volume ratio (SA/V), ratios were calculated following Narushin (2005) [20]. Eggshell thickness, eggshell weight (SW, g), and yolk weight (YW, g) were also recorded. Eggshells were first cleaned with demineralized water and then dried in an oven at 45 °C for 12 h before weighing [21]. Albumen weight (AW, g) was calculated as EW − (YW + SW). In addition, the following ratios were calculated: SW/EW (Shell %), YW/EW (Yolk %), AW/EW (Albumen %), (YW + AW)/EW (Edible %), YW/AW (Yolk/Albumen ratio).

A CR-300 Chroma Meter (Minolta Camera Co., Osaka, Japan) was used to evaluate the color of the eggshell and yolk according to the CIE-Lab color system [22], with the indexes L* (Lightness), a* (Redness), and b* (Yellowness) being measured. The colorimeter was equipped with an 8 mm measuring area and was illuminated with a pulsed Xenon arc lamp (illuminat C) at a viewing angle of 0°. Reflectance measurements were obtained at a viewing angle of 0° and the spectral component was included. The mean of six replications at the product sample surface was presented for each value. Yolk color was also assessed visually using the DSM yolk color fan (DSM, Heerlen, The Netherlands) [23].

### 2.5. Chemical Analyses

Dry matter (method 934.01), crude protein (method 979.09), ether extract (method 920.39), and ash (method 942.05) contents were determined in duplicate according to official analytical procedures [24,25]. The ash content of the shell was determined on eggshell samples to evaluate the mineral fraction.

Cholesterol content was determined using a commercial enzymatic photometric test-kit (ENZYTECTM fluid Cholesterol E8320, R-Biopharm, Melegnano, Italy) and expressed as mg per 100 g of whole egg.

### 2.6. Sensory Analyses

#### 2.6.1. Sample Preparation

The eggs were collected according to treatments and stored at 4 °C for 6 days (fresh egg category). On the day of the analysis, the eggs were kept at room temperature (20 °C) for 2 h. The samples were then placed in cold water (200 mL per egg) and boiled for 10 min from the point of boiling. The samples were then cooled by immersing them in cold water for 10 min. Once cooled, the eggs were shelled and cut into two halves along the long axis, after which they were assessed.

#### 2.6.2. Descriptive Analyses

A panel trained in sensory analysis and familiar with eggs was selected for the sensory analysis [26]. All assessments were carried out in a sensory laboratory equipped according to ISO recommendations [27]. The panel consisted of 10 judges, equally divided by gender (5 men and 5 women), aged between 30 and 40, with an average age of 33. Sessions were conducted to develop a common vocabulary and improve the ability of judges to discriminate between samples, as well as the correct use of the intensity scale. This resulted in the final list of descriptors used for the eggs consisting of 14 attributes: 4 of appearance (yolk color, yolk firmness, albumen color, yolk/albumen ratio), 2 of aroma (aroma global intensity, sulphureous aroma), 2 of taste (yolk sweetness, yolk saltiness), 2 of flavor (yolk flavor intensity, sulphureous flavor) and 4 of texture in mouth (yolk firmness, albumen firmness, yolk solubility, yolk adhesiveness).

The panel evaluated each sample in triplicate. The eggs were served on plastic plates coded with three-digit numbers, of which the order was randomized judge by judge and session by session [28].

During training and sampling, the panelist had access to unlimited water and unsalted crackers. The judges were then asked to assign a score for each descriptor, corresponding to the perceived intensity, using a 10 cm continuous non-structured scale anchored at either end by 0 (total absence of perception of the descriptor) and 10 (maximum perceived intensity of the descriptor).

### 2.7. Statistical Analyses

Data were analyzed using SAS software, version 9.4 for Windows [29]. For physical parameters, four eggs were collected from each cage at each sampling week and measured individually. Because the cage represented the experimental unit, measurements from the four eggs were averaged within the cage and sampling week before statistical analysis. Thus, one cage-by-week mean was used in the statistical analysis for each physical parameter. For chemical parameters, the edible portions of the four eggs collected from each cage at each sampling week were pooled and homogenized before analysis, resulting in one pooled sample per cage and sampling week. Data were analyzed using the PROC MIXED procedure of SAS. Dietary treatment (CON, T20, and T40), sampling week, and their interaction were included as fixed effects. Cage nested within dietary treatment was included as a random effect, with cage considered the experimental unit. Repeated measurements over sampling weeks were modeled within cage nested within dietary treatment using a compound symmetry covariance structure. Degrees of freedom were adjusted using the Kenward–Roger method. Least Squares means (LS means) were estimated for dietary treatment, sampling week, and their interaction, and pairwise comparisons were performed using Tukey’s adjustment. Data are presented as LS means ± standard error (SE), and differences were considered statistically significant at *p* < 0.05.

Data from sensory evaluation were analyzed by three-way ANOVA with samples (3), judges (10), replicates (3), and their interactions as effects (ISO). The significance of these effects was tested with F tests. Post hoc pairwise contrasts were evaluated by Duncan’s test. Data are reported as means ± standard error of the means (SEM). Differences were considered statistically significant at a *p*-value of <0.05.

## 3. Results

### 3.1. Physical Parameters

Table 2 shows the effects of FFP inclusion in the laying hen diet on the physical characteristics of the eggs. No significant differences (*p* > 0.05) were observed among dietary treatments for egg length, diameter, shape index, or weight. Similarly, shell thickness, shell weight, and shell proportion were unaffected (*p* > 0.05) by dietary treatments. No differences were observed (*p* > 0.05) in albumen and yolk weight, in the yolk to albumen ratio, or the edible weight among dietary treatments. Also, the volume, surface area, and area to volume ratio did not differ significantly (*p* > 0.05) among experimental groups.

Some differences were observed in yolk color. Yolk redness (a*) was lower (*p* < 0.05) in CON than in T20 and T40, whereas yolk yellowness (b*) was higher (*p* < 0.05) in CON than in T20 and T40. Regarding yolk color assessed using the Roche DSM Yolk Color Fan, no significant differences were observed among the experimental groups (*p* > 0.05).

Figure 1 shows the percentages of the egg components relative to the weight of the whole egg. All the considered parameters were unaffected (*p* > 0.05) by dietary treatments.

### 3.2. Chemical Parameters

The data on chemical parameters of the edible components (yolk and albumen) are shown in Table 3. No differences (*p* > 0.05) were observed among dietary treatments for dry matter, crude protein, ether extract, and ash content. There were also no differences in cholesterol content among the experimental groups. Similarly, shell ash content did not differ among the groups.

### 3.3. Sensory Profile

The F values for the aroma, taste, flavor and texture parameters of the egg sensory profile are reported in Table 4. The results indicate that the different dietary treatments affect (*p* < 0.05) the yolk color and albumen firmness. Taste and flavor were not affected (*p* > 0.05) by dietary treatment. The F values for replicates were not affected (*p* > 0.05) in all the descriptors, while the judges presented differences for all the descriptors. Differences between judges are common in sensory evaluation, due to the different use of the scale [30]. No interaction between samples × replicates and judges × replicates was observed (*p* > 0.05)—highlighted by the good reproducibility of the scores given by the panelists and the homogeneity of the samples during replicates. The occurrence of sample × judge interactions for global intensity and flavor may be attributed to differences among panelists in the use of the intensity scale rather than insufficient training, as such variability is commonly observed in sensory analysis even with highly trained panels [31,32,33].

The spider plot of the sensory profile of eggs from hens fed former foodstuff-based diets is presented in Figure 2. The attribute related to appearance, aroma, taste, flavor, and texture were comparable among groups except for yolk color (*p* < 0.05) and albumen firmness (*p* < 0.01).

## 4. Discussion

The identification of sustainable feed ingredients that comply with the concepts of the circular economy is essential for the sustainable production of the poultry sector, and one option is to use commercially processed FFPs as partial replacement of maize [34]. The data on the productive performances of laying hens fed FFP products were recently reported in the paper of Zeni et al. [35]. The present study evaluated the effects of different inclusions of former foodstuffs in the laying hen diet on physical, chemical, and sensory parameters of eggs.

The physical parameters of eggs revealed no difference among experimental groups in length, diameter, shape index, and egg weight. The same results were reported by Grigorova et al. (2023) in a study involving Lohmann brown^®^ hens fed a diet containing 20% of bread waste and 0.5% of dried marigold [36]. Moreover, Olafadehan et al. (2010) reported that inclusion of 10–30% of dried bakery waste improved egg production without affecting the egg weight [37].

The shell thickness and shell weight were unaffected by dietary treatments. The thickness of the eggshell is one of the most important indirect measurements of its resistance. The shell thickness observed in the present study was in the typical range for commercial laying hens (0.30 mm and 0.40 mm) [38,39]. The shell thickness and weight are influenced by several factors such as age, genetics, calcium metabolism, environmental, and dietary mineral supply [40].

The data about volume, surface area, and the ratio between area and volume did not differ between the three experimental groups, in line with the findings of different studies on laying hens [40,41].

Eggshell color is a trait of commercial significance, driving consumer choice. The pigments responsible for determining the brown color of eggshells are protoporphyrin IX [42]. No significant differences were observed among the groups for shell color parameters (L*, a* and b*). This finding agrees with the results of a previous study, given that eggshell color is predominantly a genetically determined trait [43].

The physical analysis regarding the yolk color reported some significant differences. Redness (a*) was significantly lower in the CON group than two treated groups. Different results were found by Rocha Gebert et al. (2020) [44] who found in laying hens fed an increasing level of biscuit residue (up to 28%) a progressive decrease in yolk redness with increasing inclusion levels.

The reduction in yolk yellowness (b*) in groups fed FFPs can be attributed to a lower dietary supply of xanthophylls resulting from the partial replacement of maize with former food [44,45]. Moreover, the increase in yolk redness values observed in the groups receiving FFPs is related to the specific types of carotenoids (lutein and zeaxanthin vs. red xanthophylls) rather than just the quantity [46].

The proximate composition and cholesterol content of the edible part of eggs are in line with data reported in the literature [47,48]. The data on proximate composition of the edible part of eggs related to dry matter, protein content, ether extract, and ash are in accordance with the study of Grigorova et al. [36]. In this study, the authors analyzed the chemical composition of egg yolk and albumen separately. They found that egg dry matter, protein content, ether extract, and ash content from hen fed diet containing 20% bread waste were similar to those of the control group. The data relating to cholesterol content did not reveal any differences between groups, and this finding is consistent with the results reported by Grigorova et al. [36].

The sensory quality of a product, including its appearance, aroma and flavor, influences consumer choice and consumption. For eggs, the color of the shell and yolk, as well as the eggs’ appearance and flavor, are key factors that influence consumer choice and preferences [49]. This consumer choice is often based on the perception that yolks that appear yellower are of better quality and have better nutritional value and taste [50]. These findings emphasize that consumers frequently lack awareness regarding the yolk color and its origins. It should be made clear that the color is not related to the nutritional value of the egg. The composition of hen diets can influence the composition and nutritional quality of eggs [51,52]. Variations in yolk color are due to the source of pigmentation, which can be either natural or synthetic, and are particularly affected by the composition of the feed included in the animal diets [53].

As shown in the data about sensory evaluation, the judges perceived the yolk color from boiled eggs from the T40 group as less intense than the others. This finding should not, therefore, be interpreted as an indication of lower product quality, but may be regarded as a consequence of the hen diet, due to the lower levels of carotenoids naturally present in their feed. It is well known that the levels of carotenoids in hen diets directly influence the color of their eggs because all carotenoids deposited in the yolk come from the dietary carotenoids. The hen stores preferentially xanthophylls (lutein and zeaxanthin) in its body fat and in the egg yolk. Color intensity and homogeneity of the egg yolk are directly dependent on the choice of dietary raw materials. FFPs were included as a partial replacement for corn, which is an important dietary source of xanthophylls. Accordingly, yolk yellowness was lower (*p* < 0.05) in eggs from hens fed with FFP diets than in those from hens fed the control diet These results are consistent with the findings obtained from the instrumental color measurements.

Moreover, the firmness of the albumen was higher (*p* < 0.01) in eggs from the control group than in eggs from groups receiving dietary intervention (T20 and T40). It is reported that firmness of the egg albumen after cooking is influenced by the structural properties and interactions of its main proteins, particularly the ovomucin–ovalbumin network. The relationship between ovomucin and ovalbumin may be affected by diets, albumen dry matter, protein content and composition, and pH values [54,55]. Even if these parameters were not investigated in the present paper, they warrant further investigation in the future to support the optimization of egg-based products in food science.

## 5. Conclusions

The present findings suggest that the inclusion of FFPs in laying hen diets may represent a potential strategy to improve feed resource efficiency and reduce food waste. The inclusion of FFPs resulted in limited changes in egg quality, although some differences were observed in yolk color and albumen firmness. These results provide preliminary evidence of the potential use of FFPs as alternative feed ingredients in laying hen diets, without negatively affecting egg quality parameters. Further studies are needed to investigate the effects of FFP inclusion in laying hen diets throughout the laying cycle and under commercial production conditions, with the aim of assessing their potential benefits for egg-based products and economic sustainability.

## Figures and Tables

**Figure 1:**
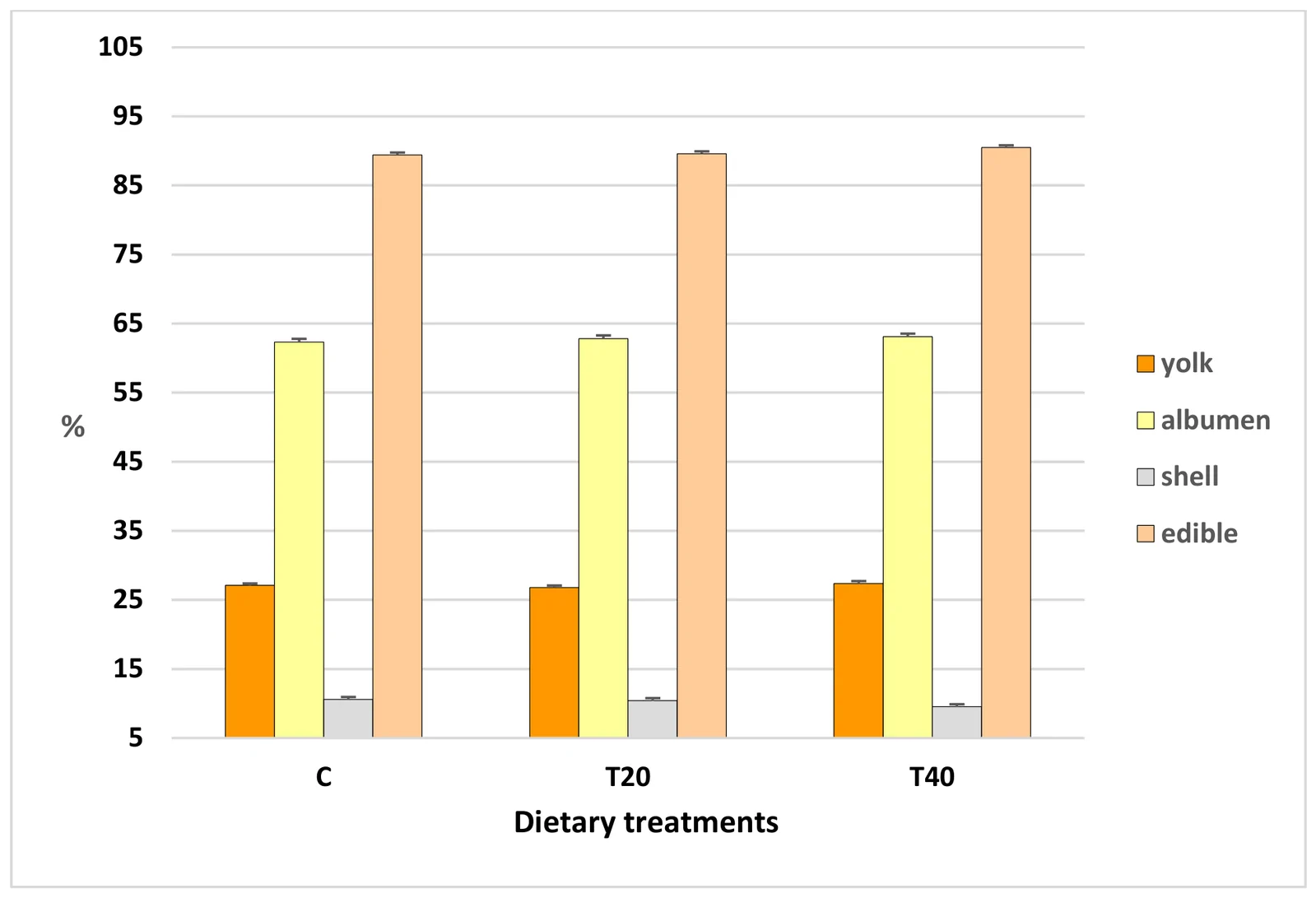
Percentage of the egg components from laying hens fed a control diet (CON) or diets containing 20% (T20) and 40% (T40) of former foodstuff products. Data are reported as LS means ± SE. The cage was the experimental unit (*n* = 5 cages/treatment).

**Figure 2:**
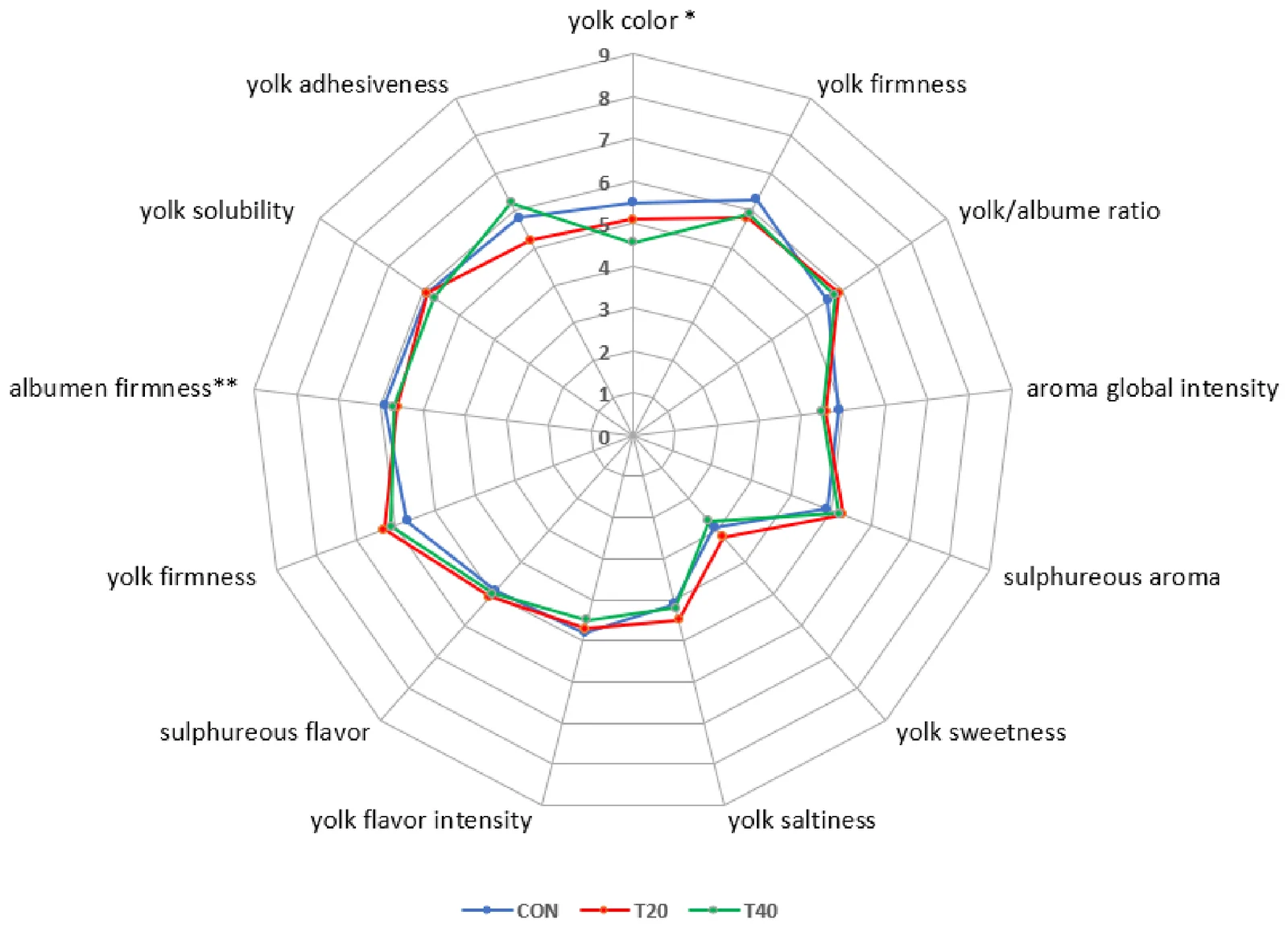
Spider plot of the sensory profile of eggs from laying hens fed a control diet (CON) or diets containing 20% (T20) and 40% (T40) former foodstuffs. * *p* < 0.05; ** *p* < 0.01.

**Table 1:** Ingredients and chemical composition of experimental diets.

Ingredients (g/kg as Fed)	CON ^1^	T20 ^1^	T40 ^1^
Corn meal	496.6	293.6	147.1
Soybean meal	197.0	159.6	162.3
Former food	0.0	200.0	400.0
Wheat	80.0	80.0	80.0
Sunflower protein	50.0	50.0	10.4
Wheat middlings	0.0	50.0	50.0
Bran	17.9	30.9	40.0
Alfalfa meal	12.2	1.5	0.0
Animal fat	43.3	29.4	1.8
Soybean oil	0.0	2.6	6.8
Calcium carbonate	88.7	89.7	89.9
Dicalcium phosphate	3.2	2.1	1.8
Sodium chloride	2.5	1.3	0.0
DL-Methionine	1.7	1.7	1.9
L-Lysine	0.3	1.1	1.3
Tryptophan	0.0	0.2	0.4
Choline chloride	1.2	1.2	1.2
Farmicol 5R-rox R 50 ^2^	0.8	0.8	0.8
BELFEED B220 ^3^	0.5	0.5	0.5
PBA HIPHOS ^4^	2.0	2.0	2.0
Mineral–vitamin premix ^5^	1.3	1.3	1.3
**Chemical composition (% as fed)**			
Dry Matter	92.48	92.31	92.19
Crude Protein	16.71	16.69	16.64
Ether extract	5.99	6.05	6.11
Crude fiber	5.01	5.07	4.57
Ash	10.49	10.94	10.77
Linoleic acid	1.60	1.60	1.60
Sugar (expressed in sucrose)	2.6	5.7	8.8
Sodium	0.17	0.17	0.17
Calcium	3.80	3.80	3.80
Phosphorous	0.42	0.42	0.38
Potassium	0.75	0.71	0.69
Chloride	0.24	0.23	0.20

^1^ CON = control diet, T20 = experimental diet with 20% former foodstuff product inclusion, T40 = experimental diet with 40% former foodstuff product inclusion. ^2^ Farmicol 5R-rox R 50: xanthophylls. ^3^ BELFEED B220: xylanase. ^4^ PBA HIPHOS: phytase IT200001MI Ricon. Reg. (EC) n°183/2005. ^5^ Mineral–vitamin premix: vitamin A, 8,000,000 I.U.; vitamin D3, 2,400,000 I.U.; vitamin E, 16,000 mg; vitamin K3, 800 mg; vitamin B1, 320 mg; vitamin B2, 2040 mg; vitamin B6, 1200 I.U.; vitamin B12, 12 mg; pantothenate, 4800 mg; niacin, 12,000 mg; folic acid, 240 mg; Fe, 15,200 mg; Mg, 50,000 mg; Zn, 40,000 mg; I, 640 mg; Se, 240 mg; Cu, 6400 mg.

**Table 2:** Physical parameters of eggs from laying hens fed a control diet (CON) or diets containing 20% (T20) and 40% (T40) of former foodstuff products.

Item	CON	T20	T40	*p*-Value ^1^		
				D	T	D × T
Length (mm)	56.50 ± 0.24	56.41 ± 0.25	56.78 ± 0.24	0.556	0.250	0.089
Diameter (mm)	44.29 ± 0.18	44.50 ± 0.18	44.28 ± 0.17	0.641	0.468	0.493
Shape index	78.42 ± 0.34	78.93 ± 0.34	78.05 ± 0.34	0.226	0.545	0.272
Shell thickness (mm)	0.34 ± 0.01	0.37 ± 0.01	0.34 ± 0.01	0.108	0.573	0.163
Egg weight (g)	62.72 ± 0.63	63.54 ± 0.64	63.00 ± 0.63	0.663	0.714	0.086
Yolk weight (g)	16.96 ± 0.24	17.01 ± 0.24	17.16 ± 0.24	0.840	0.347	0.105
Albumen weight (g)	39.11 ± 0.60	39.93 ± 0.61	39.83 ± 0.61	0.592	0.836	0.067
Shell weight (g)	6.64 ± 0.24	6.61 ± 0.24	6.01 ± 0.24	0.155	0.232	0.069
Edible (g)	56.07 ± 0.58	56.94 ± 0.59	56.99 ± 0.58	0.481	0.907	0.065
Yolk/albumen ratio	0.44 ± 0.01	0.43 ± 0.01	0.43 ± 0.01	0.821	0.299	0.075
Egg volume (cm^3^)	58.27 ± 0.63	58.78 ± 0.64	58.56 ± 0.63	0.847	0.3349	0.170
Surface area (cm^2^)	72.48 ± 0.52	72.85 ± 0.53	72.74 ± 0.52	0.878	0.3152	0.148
Area/volume ratio	1.24 ± 0.01	1.24 ± 0.01	1.24 ± 0.01	0.864	0.311	0.222
Color indexes yolk:						
L*	52.47 ± 0.35	52.01 ±0.35	51.27 ± 0.35	0.088	0.093	0.071
a*	4.72 ± 0.23 ^a^	5.46 ± 0.23 ^b^	5.79 ± 0.23 ^b^	0.016	0.098	0.425
b*	38.40 ± 0.93 ^a^	36.88 ± 0.93 ^b^	35.57 ± 0.93 ^b^	0.040	0.083	0.641
Roche Scale	12.27 ± 0.11	12.37 ± 0.11	12.32 ± 0.11	0.473	0.514	0.120
Color indexes shell						
L*	56.56 ± 0.49	55.86 ± 0.51	57.24 ± 0.49	0.207	0.981	0.183
a*	15.95 ± 0.43	16.03 ± 0.43	15.24 ± 0.43	0.390	0.091	0.159
b*	30.62 ± 0.39	30.20 ± 0.40	30.06 ± 0.40	0.571	0.063	0.821

Data are reported as LS means ± SE. The cage was the experimental unit (*n* = 5 cages/treatment). Four eggs per cage were measured at each sampling week and averaged within cage and week before analysis. ^a,b^ for *p* < 0.05. ^1^ D = diet; T = sampling week; D × T = diet × sampling week interaction.

**Table 3:** Proximate composition of edible components and shell of eggs from laying hens fed a control diet (CON) or diets containing 20% (T20) and 40% (T40) former foodstuff products.

	CON	T20	T40		*p*-Value ^1^	
				D	T	D × T
Edible egg						
Dry matter, %	26.07 ± 0.91	25.98 ± 0.91	25.40 ± 0.91	0.853	0.324	0.3536
Crude protein, %	12.59 ± 0.16	12.82 ± 0.16	12.50 ± 0.16	0.352	0.267	0.891
Ether extract, %	8.64 ± 0.30	8.93 ± 0.30	8.12 ± 0.29	0.190	0.160	0.515
Ash, %	1.32 ± 0.11	1.30 ± 0.11	1.03 ± 0.11	0.150	0.110	0.195
Cholesterol, mg/100 g	311.4 ± 9.41	313.6 ± 9.27	294.0 ± 10.48	0.757	0.447	0.727
Shell						
Ash, %	91.46 ± 0.45	91.17± 0.45	90.83 ± 0.45	0.621	0.289	0.345

Data are reported as LS means ± SE. The cage was the experimental unit (*n* = 5 cages/treatment). At each sampling week, the edible portions of four eggs from each cage were pooled to obtain one analytical sample per cage. ^1^ D = diet; T = sampling week; D × T = diet × sampling week interaction.

**Table 4:** The F values and statistical significance for the effects of samples, judges, replicates, and their interactions on sensory descriptors.

Descriptor	Samples ^1^	Judges ^1^	Replicates ^1^	S × J ^2^	S × R ^2^	J × R ^2^
Appearance						
Yellow of yolk	2.74 *	3.27 **	2.28	0.44	0.38	0.64
Yolk firmness	0.64	3.55 **	0.80	0.38	2.57	0.75
Yolk/albumen ratio	0.28	3.50 **	0.06	0.34	1.54	0.48
Aroma						
Global intensity	0.65	10.52 ***	2.77	2.12 *	0.42	1.17
Sulphureous aroma	0.36	8.28 ***	1.06	1.82	0.45	0.61
Taste						
Sweet—yolk	1.96	46.79 ***	0.99	1.84	0.53	1.88
Salty—yolk	0.51	7.53 ***	0.72	0.54	0.68	1.68
Flavor						
Flavor intensity—yolk	2.77	4.70 ***	0.47	2.34 *	0.97	0.90
Sulphureous flavor	1.19	6.72 ***	0.67	2.63 *	0.79	1.48
Texture						
Yolk firmness	0.94	5.76 ***	0.38	0.45	0.35	0.53
Albumen firmness	5.61 **	24.68 ***	3.02	3.42	0.44	1.17
Solubility—yolk	1.72	6.18 ***	1.83	2.18	0.85	1.10
Adhesivity—yolk	2.57	8.19 ***	0.88	0.85	1.56	0.95

^1^ Samples (*n* = 3), judges (*n* = 10), replicates (*n* = 3); ^2^ S × J: samples per judges; S × R: samples per replicates; J × R: judges per replicates; * for *p* < 0.05; ** for *p* < 0.01; *** for *p* < 0.001.

## Data Availability

Research data will be provided upon request.

## Abbreviations

The following abbreviations are used in this manuscript:

FFPs	Former food products
CON	Control diet
T20	Diet containing 20% former food products
T40	Diet containing 40% former food products
EW	Egg weight
D	Diameter
L	Length
V	Volume
SA	Surface area
SW	Shell weight
YW	Yolk weight
AW	Albumen weight
L*	Lightness
a*	Redness
b*	Yellowness
EdW	Edible weight
YW/AW	Yolk weight to albumen weight ratio
